# Discussion of "Tobacco and Its Effects."

**Published:** 1892-12

**Authors:** 


ARTICLE VIII.

### DISCUSSION OF "TOBACCO AND ITS EFFECTS."

Dr. Swain.—I desire to congratulate the essayist on giving us so good a paper upon this subject. I went to the books for ideas with which to open the discussion, but I found them very meager indeed; there was very little said about it, and most of that was that years ago it was considered to possess great medical virtues, but of late years the use of tobacco as a medicine has been largely discarded. I believe its curative virtue, if it has a good property, is that it is a sedative. The long continued use of tobacco is unquestionably, however, a nervous irritant, and any person who has used it for many years, either as a chewer or smoker, will, I think, concur in the statement that it becomes an irritant rather than a sedative. I question very much the statement that the excessive use of tobacco will help one mentally or physically. We have all observed in those people who have used tobacco for a number of years the conditions explained by the essayist, namely, the receding of the gums and the softening of the tooth about its neck. I can speak on this subject somewhat from experience; I used tobacco myself from childhood up to a little more than a year ago. I have chewed and I have smoked, and I am satisfied that my teeth were injured from the use of tobacco, not only in chewing from the grit which it contained causing mechanical abrasion, but in that peculiar condition which it produces of congesting the mucous surfaces which results in a disorder of the subcutaneous glands causing them to give off a diseased secretion which destroys the tooth. I don't know that I have ever observed any bad effects of nicotin or the product of combustion of smoking, on the enamel of the teeth other than to stain it; tobacco colors the teeth yellow, but the other conditions I have not noted during my experience as a practitioner of dentistry. The worst results from the use of tobacco come from the smoking of cigarettes. The combustion of the paper, especially the cheaper qualities of paper, I consider very deleterious. Just what the products are I cannot say, but the condition of the mucous membrane of the mouths in those people who are confirmed cigarette smokers gives us all the evidence that is necessary that it is very injurious.

Dr. Gilmer.—My experience leads me to the belief that tobacco tends to prevent decay of the teeth. I recall a number of instances where patients have left off for a time the use of tobacco, and in these cases almost invariably has there been a remarked increase in the tendency to decay. So frequently have I observed this that I cannot but conclude that tobacco must have a preservative effect. If this condition were only found in the mouth of former chewers we might question whether the increase in decay did arise from the lack of friction offered by the chewing process rather than by virtue contained in tobacco, but I have seen similar results following the discontinuance of smoking but not so marked.

Dr. Harlan.—I do not rise for the purpose of defending the use of tobacco, but the thing I would like to know is which are you going to do, chew gum or chew tobacco? What is there about tobacco that will cause deterioration of the teeth? Tobacco is a vegetable, cabbage is a vegetable. Cabbage is an article of diet, but it is capable of being so treated that there will be so much lactic acid as to injure the teeth. Tobacco is never treated in that way, so that you do not get the ill effects of it in chewing or smoking. Opium is a vegetable, a so-called medicinal plant, it has a great many derivatives, many of which are used in medicine as sedatives, etc. Tobacco *per se* does not injure the teeth any more than corn husks or bean shells; not half as much as beans, for the simple reason that it does not contain the constituents that micro-organisms can live upon. It is the misuse and abuse of tobacco that makes it one of the objects to which civilization is directing its eyes. The experiments which have been made with reference to smoking tobacco are successful as to its properties in that respect; but does tobacco smoke affect the teeth? If it does, it does it beneficially, that is, it will destroy the micro-organisms on which the proliferation of dental caries depends; but tobacco used in chewing is used in a bad way.

One of the reasons why people suffer from stomatitis and things of that sort is because they do not use anything that will act as a laxative, and tobacco to a great many people is a laxative, and in so far as it is a laxative it is beneficial to health. Nine-tenths of the bad smells which you encounter are not due to the supposed gases at all, but to the inaction of the ailmentary canal. If you do not bathe, or if you do not drink enough water the contents of the alimentary canal are not discharged and consequently people suffer from bad breath. They perspire from the whole glandular system; and are disagreeable to every one who comes in contact with them. Those people I recommend to take a smoke. You know what tobacco is, but the other odors comes from unknown and unseen sources that you do not know anything about.

With reference to the effects of tobacco on the teeth, there is a circle all around the necks of the teeth above the termination of the enamel that it is due to filth, that is due to the continual bathing of the mouth with the fluids, the juices that are extracted from tobacco by chewers. I have known any number of men who have chewed tobacco moderately and who are sixty-five and seventy years of age and their teeth are all sound, and I have known others whose teeth have decayed rapidly, but they would have decayed anyway.

With reference to the wearing down and mechanical abrasion, I had in my office to-day a gentleman who has eight teeth in his upper jaw and nine in his lower and he never chewed tobacco in his life, and all of these teeth have worn down until they are nearly to the gum. So that it is not an uncommon thing for the teeth to be worn down by persons who are not tobacco chewers. I don't defend the habit; I never smoked until I was twenty-five years of age and I didn't do it then from choice, but I do now and tobacco is a great satisfaction to me every summer, and it is to any man who may be walking in the woods, traveling in a boat, or be in a solitary condition. I apprehend that it is more used by people who are in the habit of thinking by themselves and who want to be undisturbed, and they use it for its sedative properties, probably, in the same way as some other people use articles of food. There is no doubt but what there are many people who eat too much pie every day, and pie is very much worse than tobacco if you eat three pieces a day, and if you eat ice cream every time you get a chance it is a very bad thing for the coats of your stomach. If you go to discussing tobacco on its merits I will put it against any vegetable or animal tissue that is used for the purpose of nutrition, because it prevents waste. A man can take a chew of tobacco, or smoke a pipe or a cigar and he can endure more, he can walk further and go longer without water to drink than the man who simply eats the same quantity by weight of oats or wheat or corn or rice or animal tissue. So I say it prevents tissue waste, and that is the reason why the whole civilized world has taken up its use. I do not discuss the question of the use of tobacco simply on account of its preservative or its non-preservative qualities on the teeth, because it is inert, it does not affect the teeth at all. There is nothing in tobacco deleterious to the teeth and micro-organisms will not pry into tobacco juice. I simply state that as a proposition which any scientific man here can disprove if he has the necessary proof. Tobacco juice will not ferment when it is mixed with saliva and if you don't have a fermentable substance you don't have anything that putresces, and so you have to leave that out of the question. There is no doubt that tobacco will stain the teeth after the enamel has been worn off; there is no doubt that it will, on account of this agency around the necks of the teeth, tend to promote an aggregation of foreign particles and large deposits of salivary calculus, a form of which is the lepothrix buccalis, and that is one of the most annoying organisms that live in the mouth. One of the greatest scientific men in dental surgery advances the theory that caries is caused by lepothrix buccalis. It is claimed that micro-organisms never produced caries in the world but on account of their location on the teeth and growing on what they were fed, that in the decomposition of the saliva in consequence of the coming in contact with it of ammoniacal products and the liberation of the elements by which they grow; and if dirt and grease, and things like that collect around them, perhaps there might be cavities. I don't advise any person to begin the use of tobacso, to chew or smoke it; I simply say from the standpoint of science that tobacco as tobacco is not injurious to the teeth at all.

Dr. Newkirk.—Having used the weed at one time myself, I think I can look at the tobacco question fairly. I believe that some of the accusations presented against it to-night are true, that on the whole it usually does exert an injurious influence, especially upon the nervous system; that it very frequently has a bad effect on the action of the heart, and that physicians frequently meet with cases where they would be very glad if they could prohibit its use. Frequently they try to have their patients use it more moderately if they cannot get them to give it up altogether. Before the discovery of America civilized man did not know anything about tobacco. We might suppose, from the eloquent plea which we have heard from Dr. Harlan, that tobacco is probably the greatest civilizer which this world possesses. See how man has advanced during the last century since the introduction of tobacco into Europe? The only fault in that theory is that the poor Indian, who has used it a great deal longer, does not seem to have advanced at all. It has had one effect upon him and another upon us. There are a great many habits in the world that are hard to account for on any reasonable grounds. I think most people form certain habits because others do, just because it is the fashion. There isn't anybody who can give a sensible reason why the ladies should be trailing their skirts along our streets at the present time. There is no reason why they should and there is every reason why they shouldn't. I saw one of the most prominet reformers in my office yesterday. "Well," I said, "fashion has you by the skirts." She said she had to fall in with the fashion, she couldn't be singular; so I suppose that is why a great many people chew tobacco and drink, and why they do a great many things, just because other people do. There is no philosophical reason for it.

I have not in my experience seen the same things which Dr. Davis [the essayist] advanced with regard to pyorrhœa. I do not remember that I have ever seen a case where I had reason to suppose the disease was produced by the use of tobacco. I have seen some of the worst cases in the mouths of those who have not used it, and I should not consider it essential in the treatment of a case to insist upon the patient leaving off. However, in chewing, masses of the weed are sometimes forced into the interspaces of the teeth, crowding the gums at the gingival margins. This acts as a mechanical local irritant and I believe has more ill effect than anything in the chemical action of the tobacco.

Dr. Ottofy.—I expected that the essayist might be able to state the reason why tobacco affects the teeth favorably. I did not think there were any who claimed that there was not some peculiar effect from tobacco upon the teeth. I do not think there are any dentists who claim that that effect is not beneficial; at least I have never heard anyone say that tobacco smoke is injurious to the teeth. We attempt to explain the cause as though it was exerted on the exterior of the tooth, but it has been for some years my opinion that tobacco smoke affects the teeth favorably through the internal circulating medium, the nicotin in the circulation in all probability exerts its influence *within* the tooth. I have always noticed that a dead tooth in a consumer's mouth is much softer and more chalky than a dead tooth in the mouth of any one else. No man can smoke and not inhale some of the smoke; in this way he gets the nicotin into the circulation very rapidly; it goes at once into the blood, being transmitted in the lungs, is circulated and brought into the tissues and the teeth, and it is there that I believe it exerts its influence. I believe that in chewing the beneficial effects are counteracted by the unfavorable effects on the soft tissues of the mouth; however even this habit is a benefit to the teeth by means of the nicotin being introduced into the teeth; it is injurious to the teeth, because chewing tobacco does contain impurities, especially molasses, sugar or grit, which will affect the gums and teeth. A habit the chewer forms, is to place the tobacco on one or the other side of the mouth on the outside of the teeth and let it rest there, which eventually results in the destruction of the gum tissue, thus overbalancing the benefit he derives from the tobacco. I would like to see the essayist continue to investigate, and determine if possible, where the beneficial effect of tobacco smoke in the mouth comes from; whether it is the effect of the tobacco upon the enamel or whether it is the effect of the tobacco in the circulation through the pulp upon the dentine.

It seems to me that whatever beneficial effect the use of tobacco has in preventing caries of the teeth is probably due to its antiseptic properties. Smoking or chewing is one of the commonest ways in which some antiseptic influence is exerted within the mouth with sufficient regularity and duration to accomplish any appreciable result, and it does not seem necessary to go any further than that to account for the ordinary beneficial results from the use of tobacco in respect to the preservation of the teeth.

Dr. Hartt.—I don't think we are looking at this subject in the right light; if tobacco preserves the teeth it does it in a very unsatisfactory manner, and we as dentists owe it to ourselves and to our brothers, to discourage the use of tobacco, because it not only destroys the beautiful appearance of the teeth, but causes a man to become careless, taking less pride in them. In addition, the man who sells the tobacco gets the money that the dentist ought to have.

In a word, let the tobacco fiend worship at the altar of his family dentist, and arm himself with a good tooth-brush, and he will then have very little excuse for continuing in a habit which is not only expensive, but very annoying to the majority of refined and cleanly people.

Dr. Brophy.—A number of years ago when the question of the preservation of the teeth by the influence of tobacco was under consideration, I had a discussion with a friend of mine who told me he had been making some experiments with a view to ascertaining whether tobacco really does preserve teeth or not; he believed that tobacco was an antiseptic and would preserve the teeth, he believed that smokers' teeth were better than the teeth of those who do not smoke, and that the teeth of chewers were better than those of people who did not chew, and he was surprised at the result of his experiments. He told me that he placed some extracted teeth in a solution of tobacco and in the course of six or eight weeks they began to disintegrate, the action of the tobacco upon them was similar to that brought about by the influence of acids. The gentleman who made these experiments was Prof. Haines of Rush Medical College. I wish Dr. Davis, if he intends to pursue this matter further, would see Dr. Haines and learn his conclusions and get the benefit of his experiments upon the teeth with tobacco. I mention his name because he is recognized everywhere as an expert upon the subject of chemistry and the results of his experiments would have a great deal of weight in settling the question.

I would say that the tobacco he used was bought at the stores where tobacco is sold to men who chew and smoke; I don't know that there is any such thing as pure tobacco. The experiments Sir Humphrey Davy conducted for over four years, with a view to procuring pure water, did not succeed. There is nothing absolutely pure; all substances are more or less adultrated.

Dr. Davis, in closing the discussion, said: As stated in the beginning of my paper, when I first took up the subject, I hoped to find some good argument in favor of tobacco, so I read, or looked over some twenty or thirty different authors, and laid just two aside who spoke favorably of the use of tobacco, and I have quoted from these two on this subject. Nothing would please me better, from the standpoint of a smoker, than to have some good authority for a favorable judgment in this matter, and I would have liked to have heard more in its favor by those who discussed the subject this evening, but only one person has spoken to any extent in its favor, and I for one should have been pleased if he had talked longer, as I wish to obtain all the favorable argument I can on this subject.

There is no particular point I can touch upon; there have been a few things said which I might criticise or emphasize, but I think on the whole the matter has had a thorough exposition.—*Dental Review.*

